# Supplementary material for: Artificial intelligence automation of echocardiographic measurements
Source: medRxiv. 2025 Mar 19:2025.03.18.25324215. Preprint. [Version 1] doi: 10.1101/2025.03.18.25324215 (PMC11957091; doi:10.1101/2025.03.18.25324215)
Supplement: Supplement 3 [file NIHPP2025.03.18.25324215v1-supplement-3.pdf]

Supplemental Materials

Supplemental Table 1: Dataset characteristics and model Performance in temporal split CSMC dataset (June 2022 – November 2024)

**Variable**	**total file**	**unique study**	**unique patient**	**Mean Sonographer Measurement (SD)**	**R^2 (95% CI)**	**ICC (95% CI)**	**MAE (95% CI)**	**Bias and limits of agreement**
**Linear Measurements**								
IVS	539	539	533	1.09 (0.26)	0.471 (0.39-0.53)	0.665 (0.56-0.74)	0.138 (0.13-0.15)	0.07: -0.28 to 0.42 cm
LVID	1,153	1,153	1,132	4.69 (0.81)	0.529 (0.47-0.58)	0.765 (0.57-0.86)	0.403 (0.38-0.42)	-0.28: -1.23 to 0.66 cm
LVPW	837	837	824	1.04 (0.22)	0.207 (0.10-0.30)	0.56 (0.38-0.68)	0.151 (0.14-0.16)	-0.09: -0.42 to 0.25 cm
Left Atrium	1,252	1,252	1,229	3.89 (0.82)	0.659 (0.60-0.71)	0.818 (0.71-0.88)	0.322 (0.31-0.34)	-0.20: -1.05 to 0.64 cm
Ascending Aorta	303	303	301	3.29 (0.47)	0.144 (-0.00-0.30)	0.594 (0.42-0.71)	0.341 (0.30-0.37)	-0.19: -0.96 to 0.59 cm
Aortic root	799	799	785	3.13 (0.47)	0.351 (0.23-0.44)	0.674 (0.49-0.78)	0.273 (0.25-0.29)	-0.18: -0.83 to 0.48 cm
RV Base	710	710	701	3.52 (0.7)	0.633 (0.55-0.70)	0.806 (0.76-0.84)	0.292 (0.27-0.32)	-0.12: -0.92 to 0.68 cm
Pulmonary Artery	142	142	138	2.19 (0.48)	-0.411 (-1.12-0.01)	0.447 (0.06-0.67)	0.469 (0.42-0.52)	-0.37: -1.20 to 0.47 cm
IVC	269	269	263	1.76 (0.53)	0.748 (0.69-0.80)	0.855 (0.77-0.9)	0.204 (0.19-0.22)	-0.11: -0.59 to 0.37 cm
**Doppler Measurements**								
TR Vmax	1,343	1,343	1,310	234.15 (53.03)	0.715 (0.66-0.76)	0.871 (0.86-0.88)	16.289 (15.37-17.19)	3.47: -51.57 to 58.51 cm/s

medRxiv preprint doi: https://doi.org/10.1101/2025.03.18.25324215; this version posted March 19, 2025. The copyright holder for this preprint (which was not certified by peer review) is the author/funder, who has granted medRxiv a license to display the preprint in perpetuity.
It is made available under a CC-BY 4.0 International license .

AV Vmax	1,234	1,234	1,212	163.31 (68.45)	0.941 (0.91-0.96)	0.97 (0.97-0.97)	9.165 (8.52-9.97)	0.96: -31.54 to 33.46 cm/s
MR Vmax	358	358	350	433.66 (101.74)	0.662 (0.52-0.79)	0.839 (0.73-0.9)	33.523 (29.32-38.38)	26.22: -77.55 to 130.00 cm/s
LVOT Vmax	961	961	947	99.34 (24.88)	0.671 (0.54-0.75)	0.851 (0.83-0.87)	8.711 (8.15-9.48)	-1.00: -28.88 to 26.88 cm/s
Lateral e'	1,364	1,364	1,340	9.39 (3.46)	0.854 (0.82-0.88)	0.927 (0.91-0.94)	0.828 (0.79-0.88)	-0.29: -2.82 to 2.25 cm/s
Septal e'	1,330	1,330	1,314	6.99 (2.52)	0.593 (0.52-0.65)	0.808 (0.66-0.88)	1.048 (0.99-1.10)	-0.77: -3.53 to 1.99 cm/s
Peak E velocity	168	168	168	81.66 (28.93)	0.765 (0.59-0.89)	0.893 (0.86-0.92)	7.806 (6.14-9.71)	1.07: -26.24 to 28.38 cm/s
E/A	108	108	108	1.25 (0.64)	0.868 (0.74-0.92)	0.936 (0.91-0.96)	0.140 (0.11-0.18)	-0.03: -0.48 to 0.42
TAPSE	981	981	975	2.09 (0.48)	0.962 (0.95-0.97)	0.981 (0.98-0.98)	0.062 (0.06-0.07)	0.00: -0.19 to 0.19 cm

IVS: Intraventricular septum, LVID: Left ventricular internal diameter, LVPW: Left ventricular posterior wall, RV Base: Right ventricular basal diameter, IVC: Inferior vena cava, TR Vmax: Tricuspid regurgitation maximum velocity, AV Vmax: Aortic valve maximum velocity, MR Vmax: Mitral regurgitation maximum velocity, LVOT Vmax: Left ventricular outflow tract maximum velocity, Lateral e': Lateral mitral annulus e' velocity, Septal e': Septal mitral annulus e' velocity, TAPSE: Tricuspid annular plane systolic excursion, CSMC: Cedars-Sinai Medical Center

medRxiv preprint doi: https://doi.org/10.1101/2025.03.18.25324215; this version posted March 19, 2025. The copyright holder for this preprint (which was not certified by peer review) is the author/funder, who has granted medRxiv a license to display the preprint in perpetuity.
It is made available under a CC-BY 4.0 International license .

Supplemental Table 2: External dataset characteristics and model Performance in Stanford Health Care (Dataset with image-level ground truth)

Variable	total file	unique study	unique patient	Mean Sonographer Measurement (SD)	R^2 (95% CI)	ICC (95% CI)	MAE (95% CI)	Bias and limits of agreement
**Linear Measurements**								
IVS	96	96	94	1.02 (0.21)	0.617 (0.46-0.72)	0.78 (0.58-0.87)	0.106 (0.09-0.12)	0.06: -0.16 to 0.29
LVID	129	129	129	4.69 (0.78)	0.853 (0.76-0.91)	0.922 (0.89-0.95)	0.210 (0.17-0.25)	-0.08: -0.64 to 0.49
LVPW	55	55	54	1.01 (0.21)	0.712 (0.50-0.82)	0.826 (0.72-0.89)	0.093 (0.08-0.11)	-0.02: -0.23 to 0.20
Left Atrium	35	35	35	4.06 (0.72)	0.403 (-0.30-0.70)	0.742 (0.47-0.87)	0.410 (0.30-0.53)	0.27: -0.66 to 1.20
Ascending Aorta	77	77	77	3.5 (0.47)	0.450 (0.08-0.66)	0.757 (0.42-0.88)	0.281 (0.24-0.33)	0.21: -0.35 to 0.76
Aortic root	32	32	32	3.25 (0.47)	0.422 (-0.14-0.66)	0.737 (0.24-0.9)	0.286 (0.22-0.36)	0.23: -0.28 to 0.74
RV Base	60	60	60	3.5 (0.6)	0.800 (0.64-0.90)	0.896 (0.83-0.94)	0.184 (0.14-0.23)	-0.04: -0.55 to 0.48
Pulmonary Artery	13	13	13	3.31 (0.78)	0.414 (-1.92-0.71)	0.714 (0.11-0.91)	0.489 (0.33-0.66)	0.39: -0.43 to 1.21
IVC	67	67	67	1.76 (0.5)	0.738 (0.57-0.84)	0.85 (0.62-0.93)	0.185 (0.15-0.23)	-0.14: -0.55 to 0.27
**Doppler Measurements**								

medRxiv preprint doi: https://doi.org/10.1101/2025.03.18.25324215; this version posted March 19, 2025. The copyright holder for this preprint (which was not certified by peer review) is the author/funder, who has granted medRxiv a license to display the preprint in perpetuity.
It is made available under a CC-BY 4.0 International license .

TR Vmax	425	425	423	258.41 (46.07)	0.786 (0.73-0.84)	0.913 (0.9-0.93)	15.392 (14.00-16.80)	5.49: -34.83 to 45.82
AV Vmax	320	320	320	170.62 (67.04)	0.943 (0.91-0.97)	0.977 (0.97-0.98)	10.407 (9.14-11.84)	4.31: -25.99 to 34.61
MR Vmax	89	89	89	492.54 (50.08)	0.323 (-0.14-0.60)	0.796 (0.63-0.88)	29.922 (24.45-36.16)	14.96: -59.82 to 89.73
LVOT Vmax	304	304	304	101.22 (25.56)	0.808 (0.71-0.87)	0.915 (0.88-0.94)	7.886 (7.01-8.82)	-3.69: -24.38 to 16.99
Lateral e'	559	559	559	9.3 (3.32)	0.696 (0.62-0.76)	0.845 (0.82-0.87)	1.167 (1.05-1.29)	-0.27: -3.82 to 3.29
Septal e'	640	640	639	6.94 (2.51)	0.461 (0.33-0.57)	0.735 (0.59-0.82)	1.228 (1.12-1.33)	-0.81: -4.05 to 2.43
Peak E velocity	71	71	71	84.35 (26.73)	0.810 (0.56-0.94)	0.911 (0.74-0.96)	6.796 (4.89-9.24)	6.64: -11.93 to 25.21
E/A	54	54	54	1.19 (0.79)	0.365 (0.10-0.94)	0.556 (0.34-0.72)	0.215 (0.09-0.39)	0.07: -1.14 to 1.28
TAPSE	254	254	254	2.28 (0.5)	0.512 (0.17-0.77)	0.781 (0.72-0.83)	0.148 (0.11-0.19)	0.09: -0.58 to 0.75

For a detailed explanation of echocardiography parameter abbreviations, refer to Supplemental Table 1. SHC: Stanford HealthCare

medRxiv preprint doi: https://doi.org/10.1101/2025.03.18.25324215; this version posted March 19, 2025. The copyright holder for this preprint (which was not certified by peer review) is the author/funder, who has granted medRxiv a license to display the preprint in perpetuity.
It is made available under a CC-BY 4.0 International license .

Supplemental Table 3: External dataset characteristics and model Performance in Stanford Health Care (Dataset with study-level ground truth cohort)

Variable	total file	unique study	unique patient	Mean Sonographer Measurement (SD)	R^2 (95% CI)	ICC (95% CI)	MAE (95% CI)	Bias and limits of agreement
**Linear Measurements**								
IVS	382	382	377	1.05 (0.25)	0.384 (0.06-0.60)	0.665 (0.57-0.74)	0.124 (0.11-0.14)	0.06: -0.16 to 0.29
LVID	666	666	661	4.73 (0.78)	0.754 (0.70-0.80)	0.876 (0.81-0.91)	0.275 (0.26-0.30)	-0.08: -0.65 to 0.49
LVPW	368	368	364	1.04 (0.21)	0.381 (0.26-0.48)	0.626 (0.54-0.69)	0.127 (0.12-0.14)	-0.01: -0.23 to 0.21
Left Atrium	290	279	279	4.06 (0.81)	0.609 (0.49-0.70)	0.806 (0.75-0.85)	0.372 (0.33-0.41)	0.27: -0.66 to 1.20
Ascending Aorta	247	237	235	3.38 (0.47)	0.089 (-0.20-0.31)	0.556 (0.46-0.64)	0.343 (0.31-0.38)	0.21: -0.34 to 0.76
Aortic root	377	375	374	3.31 (0.48)	0.496 (0.33-0.62)	0.732 (0.68-0.78)	0.237 (0.21-0.26)	0.23: -0.28 to 0.74
RV Base	309	309	306	3.35 (0.65)	0.483 (0.34-0.60)	0.729 (0.56-0.82)	0.320 (0.28-0.36)	-0.03: -0.56 to 0.49
Pulmonary Artery	13	13	13	3.31 (0.78)	0.414 (-1.94-0.71)	0.714 (0.11-0.91)	0.489 (0.34-0.66)	0.39: -0.43 to 1.21
IVC	139	139	138	1.76 (0.46)	0.634 (0.48-0.75)	0.787	0.197 (0.17-0.23)	-0.14: -0.55 to 0.27

medRxiv preprint doi: https://doi.org/10.1101/2025.03.18.25324215; this version posted March 19, 2025. The copyright holder for this preprint (which was not certified by peer review) is the author/funder, who has granted medRxiv a license to display the preprint in perpetuity.
It is made available under a CC-BY 4.0 International license .

						(0.65-0.86)		
**Doppler Measurements**								
TR Vmax	672	672	667	258.26 (49.74)	0.743 (0.68-0.79)	0.883 (0.85-0.91)	17.313 (15.96-18.72)	2.23: -35.95 to 40.41
AV Vmax	467	467	463	181.33 (78.03)	0.881 (0.80-0.94)	0.936 (0.92-0.95)	13.141 (11.14-15.36)	3.41: -22.80 to 29.62
MR Vmax	115	115	115	492.6 (50.76)	0.467 (0.16-0.66)	0.792 (0.68-0.86)	27.195 (23.08-31.55)	17.46: -49.41 to 84.32
LVOT Vmax	358	358	356	102.34 (26.51)	0.757 (0.65-0.83)	0.889 (0.86-0.91)	8.631 (7.64-9.69)	-3.65: -24.05 to 16.74
Lateral e'	607	607	605	9.15 (3.33)	0.678 (0.60-0.75)	0.838 (0.81-0.86)	1.173 (1.06-1.29)	-0.24: -3.81 to 3.32
Septal e'	685	685	683	6.89 (2.48)	0.454 (0.33-0.56)	0.735 (0.59-0.82)	1.218 (1.12-1.32)	-0.80: -4.04 to 2.44
Peak E velocity	153	153	152	79.41 (24.74)	0.822 (0.67-0.91)	0.913 (0.88-0.94)	6.604 (5.43-8.00)	6.64: -11.93 to 25.21
E/A	151	151	150	1.25 (0.66)	0.565 (0.32-0.86)	0.737 (0.65-0.8)	0.186 (0.13-0.25)	0.07: -1.14 to 1.28
TAPSE	257	257	257	2.28 (0.5)	0.517 (0.17-0.78)	0.783 (0.72-0.83)	0.148 (0.11-0.19)	0.09: -0.58 to 0.75

For a detailed explanation of echocardiography parameter abbreviations, refer to Supplemental Table 1. SHC: Stanford HealthCare

medRxiv preprint doi: https://doi.org/10.1101/2025.03.18.25324215; this version posted March 19, 2025. The copyright holder for this preprint (which was not certified by peer review) is the author/funder, who has granted medRxiv a license to display the preprint in perpetuity.
It is made available under a CC-BY 4.0 International license .

Supplemental Table 4: Evaluation of Deep Learning Model Performance by Predicted Image Quality for Echocardiographic Parameters (CSMC Held-Out Dataset)

Variable	Image Quality	N	Sonographer Measurements	EchoNet-Measurement	R2	MAE (95%CI)	p-value	MAE Difference
**Linear Measurements**								
IVS	Low	1534	1.14 (0.26)	1.11 (0.19)	0.25	0.162 (0.15-0.17)	<0.0001	0.02
	High	8572	1.12 (0.27)	1.1 (0.2)	0.5	0.139 (0.14-0.14)		
LVID	Low	3324	3.58 (1.05)	3.62 (0.83)	0.56	0.520 (0.50-0.54)	<0.0001	0.16
	High	20328	3.73 (1.13)	3.76 (1.02)	0.79	0.365 (0.36-0.37)		
LVPW	Low	1679	1.1 (0.22)	1.17 (0.21)	-0.04	0.169 (0.16-0.18)	<0.0001	0.03
	High	10630	1.09 (0.23)	1.13 (0.19)	0.35	0.138 (0.14-0.14)		
Left Atrium	Low	1230	3.71 (0.76)	3.65 (0.68)	0.49	0.400 (0.38-0.42)	<0.0001	0.10
	High	7786	3.86 (0.78)	3.84 (0.71)	0.72	0.301 (0.29-0.31)		
Ascending Aorta	Low	1972	3.36 (0.55)	3.35 (0.55)	0.17	0.323 (0.31-0.34)	0.01	0.02
	High	4741	3.19 (0.51)	3.16 (0.47)	0.3	0.301 (0.29-0.31)		
Aortic root	Low	2083	3.22 (0.47)	3.3 (0.58)	-0.22	0.305 (0.29-0.32)	<0.0001	0.08
	High	8003	3.18 (0.46)	3.22 (0.43)	0.48	0.229 (0.22-0.23)		
RV Base	Low	1941	3.61 (0.68)	3.57 (0.61)	0.4	0.387 (0.37-0.40)	<0.0001	0.06

medRxiv preprint doi: https://doi.org/10.1101/2025.03.18.25324215; this version posted March 19, 2025. The copyright holder for this preprint (which was not certified by peer review) is the author/funder, who has granted medRxiv a license to display the preprint in perpetuity.
It is made available under a CC-BY 4.0 International license .

	High	7491	3.67 (0.72)	3.66 (0.63)	0.63	0.328 (0.32-0.33)		
Pulmonary Artery	Low	403	2.07 (0.56)	2.15 (0.43)	0.31	0.364 (0.34-0.39)	<0.0001	0.10
	High	632	2.04 (0.75)	2.03 (0.49)	0.38	0.464 (0.44-0.49)		
IVC	Low	1256	1.64 (0.46)	1.63 (0.37)	0.32	0.288 (0.27-0.30)	<0.0001	0.07
	High	2051	1.71 (0.5)	1.68 (0.43)	0.63	0.217 (0.21-0.23)		
**Doppler Measurements**								
AV Vmax	Low	6238	122.46 (39.23)	132.92 (41.54)	0.67	14.282 (13.85-14.72)	<0.0001	1.93
	High	17793	133.44 (37.05)	142.27 (39.62)	0.72	12.353 (12.13-12.58)		
TR Vmax	Low	27934	251.99 (58.15)	244.43 (65.08)	0.51	30.313 (29.98-30.64)	<0.0001	5.77
	High	85820	249.0 (57.95)	249.07 (68.29)	0.7	24.540 (24.41-24.67)		
MR Vmax	Low	3730	412.13 (109.39)	357.32 (128.76)	0.39	62.852 (60.99-64.74)	<0.0001	21.53
	High	4595	451.13 (105.62)	428.57 (125.03)	0.66	41.322 (39.99-42.66)		
Septal e'	Low	1953	7.42 (2.68)	7.23 (2.7)	0.77	0.782 (0.74-0.83)	<0.0001	0.08
	High	16426	7.22 (2.59)	7.38 (2.54)	0.81	0.701 (0.69-0.71)		
Lateral e'	Low	8454	9.69 (3.5)	9.37 (3.39)	0.82	0.903 (0.88-0.93)	<0.0001	0.08
	High	40572	9.11 (3.29)	9.34 (3.18)	0.84	0.820 (0.81-0.83)		
LVOT Vmax	Low	1698	96.99 (31.3)	105.3 (31.76)	0.53	16.153 (15.49-16.83)	0.29	0.68
	High	1441	102.12 (49.52)	114.98 (54.78)	0.7	15.468 (14.41-16.68)		
E velocity	Low	9417	84.45 (31.44)	86.15 (30.84)	0.81	9.160 (8.95-9.37)	<0.0001	1.58

medRxiv preprint doi: https://doi.org/10.1101/2025.03.18.25324215; this version posted March 19, 2025. The copyright holder for this preprint (which was not certified by peer review) is the author/funder, who has granted medRxiv a license to display the preprint in perpetuity.
It is made available under a CC-BY 4.0 International license .

	High	17665	87.33 (28.41)	88.73 (29.06)	0.84	7.582 (7.46-7.71)		
E/A	Low	9418	1.32 (0.76)	1.31 (0.71)	0.73	0.213 (0.21-0.22)	0.50	0.00
	High	17666	1.5 (0.8)	1.46 (0.76)	0.79	0.211 (0.21-0.22)		
TAPSE	Low	2030	1.89 (0.53)	1.86 (0.53)	0.97	0.056 (0.05-0.06)	0.06	0.01
	High	24042	1.92 (0.55)	1.9 (0.54)	0.94	0.051 (0.05-0.05)		

The evaluation of the deep learning model's performance in predicting echocardiographic measurements, stratified by predicted image quality (Low vs High) by image-quality deep-learning (DL) model. For each echocardiographic parameter, the mean absolute error (MAE) with 95% confidence intervals, correlation coefficient, and p-value based on MAE values in each group are reported. For a detailed explanation of echocardiography parameter abbreviations, refer to Supplemental Table 1.

medRxiv preprint doi: https://doi.org/10.1101/2025.03.18.25324215; this version posted March 19, 2025. The copyright holder for this preprint (which was not certified by peer review) is the author/funder, who has granted medRxiv a license to display the preprint in perpetuity.
It is made available under a CC-BY 4.0 International license .

Supplemental Table 5: Evaluation of Deep Learning Model Performance by Patient Sex (CSMC Held-Out Dataset)

Variable	Sex	N	Sonographer Measurements	DL-model Measurements	R2	MAE (95%CI)	p-value	MAE Difference
**Linear Measurements**								
IVS	Male	5778	1.16 (0.26)	1.14 (0.19)	0.44	0.145 (0.14-0.15)	0.06	0.005
	Female	4328	1.08 (0.27)	1.05 (0.19)	0.48	0.140 (0.14-0.14)		
LVID	Male	13419	3.92 (1.15)	3.95 (1.02)	0.76	0.397 (0.39-0.40)	<0.0001	0.025
	Female	10233	3.43 (1.02)	3.46 (0.89)	0.73	0.372 (0.37-0.38)		
LVPW	Male	7004	1.12 (0.22)	1.17 (0.19)	0.23	0.146 (0.14-0.15)	<0.0001	0.009
	Female	5305	1.05 (0.23)	1.08 (0.19)	0.34	0.137 (0.13-0.14)		
Left Atrium	Male	4912	4.02 (0.77)	3.99 (0.7)	0.64	0.332 (0.32-0.34)	<0.0001	0.039
	Female	4104	3.63 (0.74)	3.59 (0.66)	0.70	0.293 (0.28-0.30)		
Ascending Aorta	Male	3904	3.37 (0.52)	3.33 (0.49)	0.19	0.321 (0.31-0.33)	<0.0001	0.032
	Female	2809	3.07 (0.49)	3.06 (0.48)	0.25	0.289 (0.28-0.30)		
Aortic root	Male	5718	3.36 (0.44)	3.4 (0.45)	0.23	0.250 (0.24-0.26)	0.04	0.012
	Female	4368	2.96 (0.39)	3.01 (0.4)	0.10	0.238 (0.23-0.25)		
RV Base	Male	5289	3.83 (0.7)	3.81 (0.6)	0.55	0.349 (0.34-0.36)	<0.0001	0.02
	Female	4143	3.44 (0.68)	3.42 (0.58)	0.56	0.329 (0.32-0.34)		
Pulmonary Artery	Male	485	2.1 (0.65)	2.14 (0.46)	0.34	0.401 (0.37-0.43)	0.04	0.045

medRxiv preprint doi: https://doi.org/10.1101/2025.03.18.25324215; this version posted March 19, 2025. The copyright holder for this preprint (which was not certified by peer review) is the author/funder, who has granted medRxiv a license to display the preprint in perpetuity.
It is made available under a CC-BY 4.0 International license .

	Female	550	2.01 (0.7)	2.02 (0.47)	0.37	0.446 (0.42-0.47)		
IVC	Male	1944	1.75 (0.49)	1.72 (0.41)	0.51	0.252 (0.24-0.26)	0.02	0.019
	Female	1363	1.59 (0.47)	1.58 (0.39)	0.52	0.233 (0.22-0.25)		
**Doppler Measurements**								
AV Vmax	Male	13345	127.1 (37.36)	136.26 (40.13)	0.67	12.938 (12.65-13.23)	0.36	0.19
	Female	10686	134.96 (38.2)	144.32 (40.15)	0.74	12.748 (12.47-13.02)		
TR Vmax	Male	60209	246.7 (56.13)	242.47 (65.46)	0.6	26.753 (26.57-26.94)	<0.0001	1.69
	Female	53545	253.14 (59.88)	254.07 (69.3)	0.7	25.064 (24.89-25.25)		
MR Vmax	Male	4586	425.57 (105.89)	381.33 (127.24)	0.45	55.655 (54.07-57.25)	<0.0001	10.44
	Female	3739	443.57 (112.04)	415.43 (134.34)	0.65	45.220 (43.65-46.80)		
Septal e'	Male	10220	7.19 (2.51)	7.3 (2.49)	0.79	0.706 (0.69-0.72)	0.54	0.71
	Female	8159	7.3 (2.7)	7.44 (2.64)	0.82	0.714 (0.69-0.73)		
Lateral e'	Male	27053	9.37 (3.3)	9.5 (3.16)	0.83	0.839 (0.83-0.85)	0.25	0.84
	Female	21973	9.02 (3.37)	9.16 (3.28)	0.84	0.828 (0.81-0.84)		
LVOT Vmax	Male	1870	94.75 (33.93)	105.3 (34.82)	0.63	15.323 (14.71-15.95)	0.05	1.28
	Female	1269	106.12 (48.36)	116.29 (54.37)	0.65	16.599 (15.43-17.97)		
E velocity	Male	15517	83.66 (28.36)	84.91 (28.61)	0.81	8.051 (7.91-8.20)	0.10	0.19
	Female	11565	89.9 (30.66)	91.76 (30.71)	0.84	8.238 (8.07-8.41)		
E/A	Male	15519	1.49 (0.82)	1.45 (0.77)	0.76	0.226 (0.22-0.23)	<0.0001	0.03
	Female	11565	1.37 (0.75)	1.35 (0.71)	0.79	0.192 (0.19-0.20)		

medRxiv preprint doi: https://doi.org/10.1101/2025.03.18.25324215; this version posted March 19, 2025. The copyright holder for this preprint (which was not certified by peer review) is the author/funder, who has granted medRxiv a license to display the preprint in perpetuity.
It is made available under a CC-BY 4.0 International license .

TAPSE	Male	14312	1.9 (0.58)	1.87 (0.56)	0.92	0.054 (0.05-0.06)	<0.0001	0.006
	Female	11760	1.93 (0.51)	1.91 (0.51)	0.98	0.048 (0.05-0.05)		

MAE: mean absolute error, DL: Deep learning. For a detailed explanation of echocardiography parameter abbreviations, refer to Supplemental Table 1.

Supplemental Table 6: Evaluation of Deep Learning Model Performance by a History of Atrial Fibrillation (CSMC Held-Out Dataset)

Variable	AF History	N	Sonographer Measurement	EchoNet-Measurement	R2	MAE (95%CI)	p-value	MAE Difference
**Linear Measurements**								
IVS	No AF	9591	1.12 (0.27)	1.1 (0.2)	0.46	0.143 (0.14-0.15)	0.05	0.01
	AF	515	1.17 (0.27)	1.14 (0.2)	0.54	0.131 (0.12-0.14)		
LVID	No AF	22460	3.7 (1.12)	3.73 (0.99)	0.76	0.387 (0.38-0.39)	0.31	0.01
	AF	1192	3.82 (1.26)	3.83 (1.1)	0.83	0.375 (0.36-0.40)		
LVPW	No AF	11711	1.09 (0.23)	1.13 (0.19)	0.29	0.142 (0.14-0.14)	0.99	0.00
	AF	598	1.14 (0.23)	1.18 (0.2)	0.34	0.142 (0.13-0.15)		
Left Atrium	No AF	8612	3.82 (0.77)	3.79 (0.7)	0.69	0.310 (0.30-0.32)	<0.0001	0.09
	AF	404	4.27 (0.75)	4.22 (0.69)	0.46	0.396 (0.36-0.43)		
Ascending Aorta	No AF	6375	3.24 (0.53)	3.21 (0.5)	0.28	0.307 (0.30-0.32)	0.47	0.01
	AF	338	3.29 (0.5)	3.28 (0.51)	0.09	0.320 (0.28-0.36)		
Aortic root	No AF	9592	3.18 (0.46)	3.23 (0.47)	0.33	0.244 (0.24-0.25)	0.10	0.02
	AF	494	3.24 (0.49)	3.28 (0.5)	0.32	0.266 (0.24-0.29)		

medRxiv preprint doi: https://doi.org/10.1101/2025.03.18.25324215; this version posted March 19, 2025. The copyright holder for this preprint (which was not certified by peer review) is the author/funder, who has granted medRxiv a license to display the preprint in perpetuity.
It is made available under a CC-BY 4.0 International license .

RV Base	No AF	8956	3.65 (0.71)	3.63 (0.62)	0.58	0.339 (0.33-0.35)	0.07	0.03
	AF	476	3.85 (0.78)	3.82 (0.63)	0.61	0.365 (0.34-0.39)		
Pulmonary Artery	No AF	980	2.03 (0.67)	2.05 (0.46)	0.35	0.425 (0.40-0.45)	0.90	0.01
	AF	55	2.54 (0.65)	2.52 (0.36)	0.28	0.419 (0.33-0.52)		
IVC	No AF	3139	1.68 (0.48)	1.66 (0.41)	0.52	0.243 (0.24-0.25)	0.39	0.02
	AF	168	1.74 (0.59)	1.65 (0.41)	0.58	0.259 (0.22-0.30)		
**Doppler Measurements**								
AV Vmax	No AF	22920	130.89 (37.68)	140.16 (40.16)	0.7	12.852 (12.65-13.06)	0.96	0.02
	AF	1111	124.4 (42.46)	133.33 (43.28)	0.77	12.876 (11.97-13.80)		
TR Vmax	No AF	107332	249.06 (57.93)	246.95 (67.43)	0.65	25.973 (25.84-26.11)	0.35	0.27
	AF	6422	260.99 (58.31)	264.39 (67.31)	0.67	25.705 (25.19-26.22)		
MR Vmax	No AF	7899	433.74 (109.67)	397.04 (132.19)	0.56	50.705 (49.57-51.88)	0.05	5.14
	AF	426	432.1 (97.14)	389.32 (119.37)	0.32	55.848 (50.54-61.26)		
Septal e'	No AF	17608	7.27 (2.61)	7.39 (2.57)	0.8	0.713 (0.70-0.73)	0.01	0.08
	AF	771	6.6 (2.19)	6.67 (2.09)	0.83	0.630 (0.59-0.68)		
Lateral e'	No AF	47021	9.21 (3.34)	9.34 (3.23)	0.83	0.836 (0.83-0.85)	0.09	0.04
	AF	2005	9.3 (3.25)	9.5 (3.04)	0.85	0.795 (0.75-0.84)		
LVOT Vmax	No AF	2931	100.07 (41.55)	110.29 (44.51)	0.69	15.724 (15.12-16.36)	0.18	1.73
	AF	208	89.14 (25.35)	102.01 (37.22)	-0.75	17.450 (14.21-21.88)		

medRxiv preprint doi: https://doi.org/10.1101/2025.03.18.25324215; this version posted March 19, 2025. The copyright holder for this preprint (which was not certified by peer review) is the author/funder, who has granted medRxiv a license to display the preprint in perpetuity.
It is made available under a CC-BY 4.0 International license .

E velocity	No AF	25920	86.07 (29.44)	87.61 (29.62)	0.83	8.120 (8.01-8.23)	0.34	0.26
	AF	1162	91.98 (31.01)	92.9 (31.3)	0.8	8.379 (7.76-9.02)		
E/A	No AF	25922	1.41 (0.78)	1.39 (0.74)	0.78	0.207 (0.20-0.21)	<0.0001	0.11
	AF	1162	1.94 (0.91)	1.89 (0.87)	0.67	0.315 (0.29-0.34)		
TAPSE	No AF	24673	1.93 (0.55)	1.91 (0.54)	0.94	0.051 (0.05-0.05)	0.47	0.003
	AF	1399	1.62 (0.49)	1.6 (0.49)	0.97	0.054 (0.05-0.06)		

medRxiv preprint doi: https://doi.org/10.1101/2025.03.18.25324215; this version posted March 19, 2025. The copyright holder for this preprint (which was not certified by peer review) is the author/funder, who has granted medRxiv a license to display the preprint in perpetuity.
It is made available under a CC-BY 4.0 International license .

medRxiv preprint doi: https://doi.org/10.1101/2025.03.18.25324215; this version posted March 19, 2025. The copyright holder for this preprint (which was not certified by peer review) is the author/funder, who has granted medRxiv a license to display the preprint in perpetuity.
It is made available under a CC-BY 4.0 International license .

MAE: mean absolute error, DL: Deep learning. For a detailed explanation of echocardiography parameter abbreviations, refer to Supplemental Table 1.

medRxiv preprint doi: https://doi.org/10.1101/2025.03.18.25324215; this version posted March 19, 2025. The copyright holder for this preprint (which was not certified by peer review) is the author/funder, who has granted medRxiv a license to display the preprint in perpetuity.
It is made available under a CC-BY 4.0 International license .

Supplemental Table 7: Categorization of Manually Reviewed Images with Measurement Discrepancies by Echocardiographic Parameters

	Absolute Error 90% tile	Category 1	Category 2	Category 3	Category 4
**Linear Measurements**					
IVS	0.302	4	1	5	0
LVID	0.889	5	3	0	2
LVPW	0.299	7	2	0	1
Left Atrium	0.694	4	2	3	1
Ascending Aorta	0.689	7	3	0	0
Aortic root	0.534	7	0	1	2
RV Base	0.735	6	2	2	0
Pulmonary Artery	0.88	5	1	4	0
IVC	0.522	6	0	3	1
**Doppler Measurements**					
TR Vmax	54.292	6	0	1	3
AV Vmax	28.229	3	1	5	1
MR Vmax	134.394	6	3	0	1
LVOT Vmax	31.04	5	1	3	1
Lateral e'	1.741	4	1	4	1
Septal e'	2.176	8	0	2	0
Peak E vel	17.462	3	0	7	0
E/A	0.464	4	0	5	1
TAPSE	0.102	1	0	9	0
Total		91	20	54	15

A subset of 180 images (randomly selected 10 images from each measurement variable) from CSMC held-out test cohort dataset with absolute differences between sonographer measurement and deep-learning measurement in the 90th percentile or higher was manually reviewed and categorized as follow; **Category 1**: Sonographer annotations were preferred over automatic measurements by the deep-learning model. **Category 2**: Deep-learning model measurements were preferred over sonographer annotations. **Category 3**: Both sonographer annotations and deep-learning model predictions were

medRxiv preprint doi: https://doi.org/10.1101/2025.03.18.25324215; this version posted March 19, 2025. The copyright holder for this preprint (which was not certified by peer review) is the author/funder, who has granted medRxiv a license to display the preprint in perpetuity.
It is made available under a CC-BY 4.0 International license .

clinically acceptable but demonstrated measurement variability. **Category 4**: Echocardiographic images were severely noisy or blurred, unsuitable for deep-learning-based measurements. For a detailed explanation of echocardiography parameter abbreviations, refer to Supplemental Table 1.

medRxiv preprint doi: https://doi.org/10.1101/2025.03.18.25324215; this version posted March 19, 2025. The copyright holder for this preprint (which was not certified by peer review) is the author/funder, who has granted medRxiv a license to display the preprint in perpetuity.
It is made available under a CC-BY 4.0 International license .

Supplemental Figure 1: Representative images of quality control model dataset

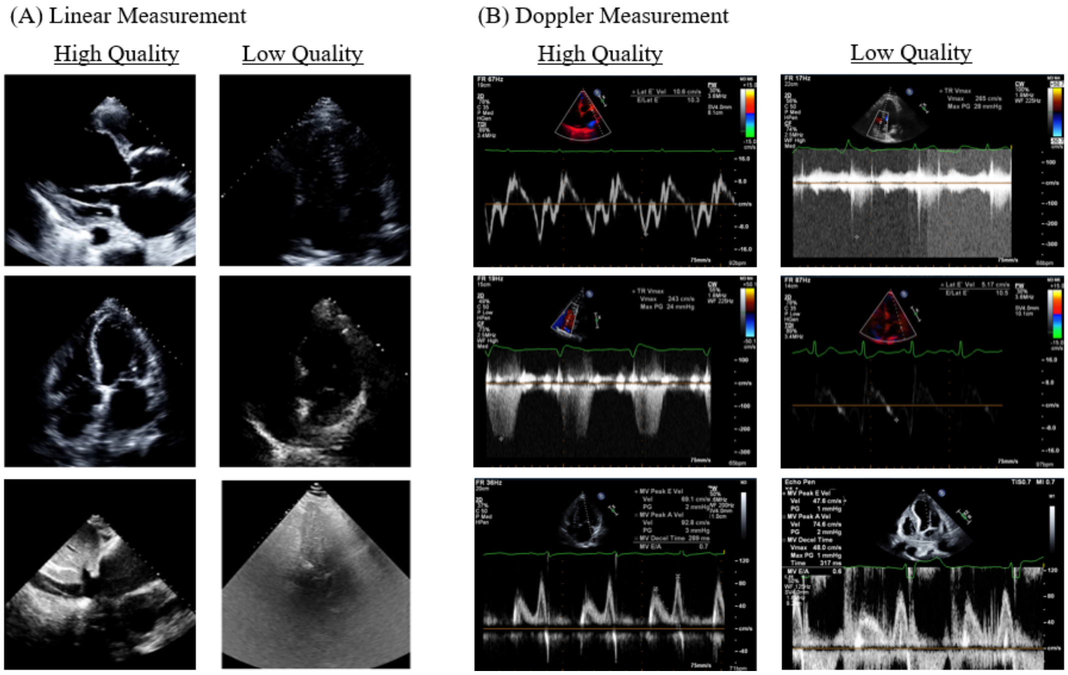

Representative images demonstrating variations in image quality within the quality control model dataset. High-quality images (left) and low-quality images (right) with blur or noise in the linear measurement model (A) and Doppler imaging models (B).

medRxiv preprint doi: https://doi.org/10.1101/2025.03.18.25324215; this version posted March 19, 2025. The copyright holder for this preprint (which was not certified by peer review) is the author/funder, who has granted medRxiv a license to display the preprint in perpetuity.
It is made available under a CC-BY 4.0 International license .

Supplemental Figure 2: Correlation between Deep Learning Model and Sonographer Annotations for Echocardiographic Linear Measurements of B-mode Classified by Predicted Image-Quality

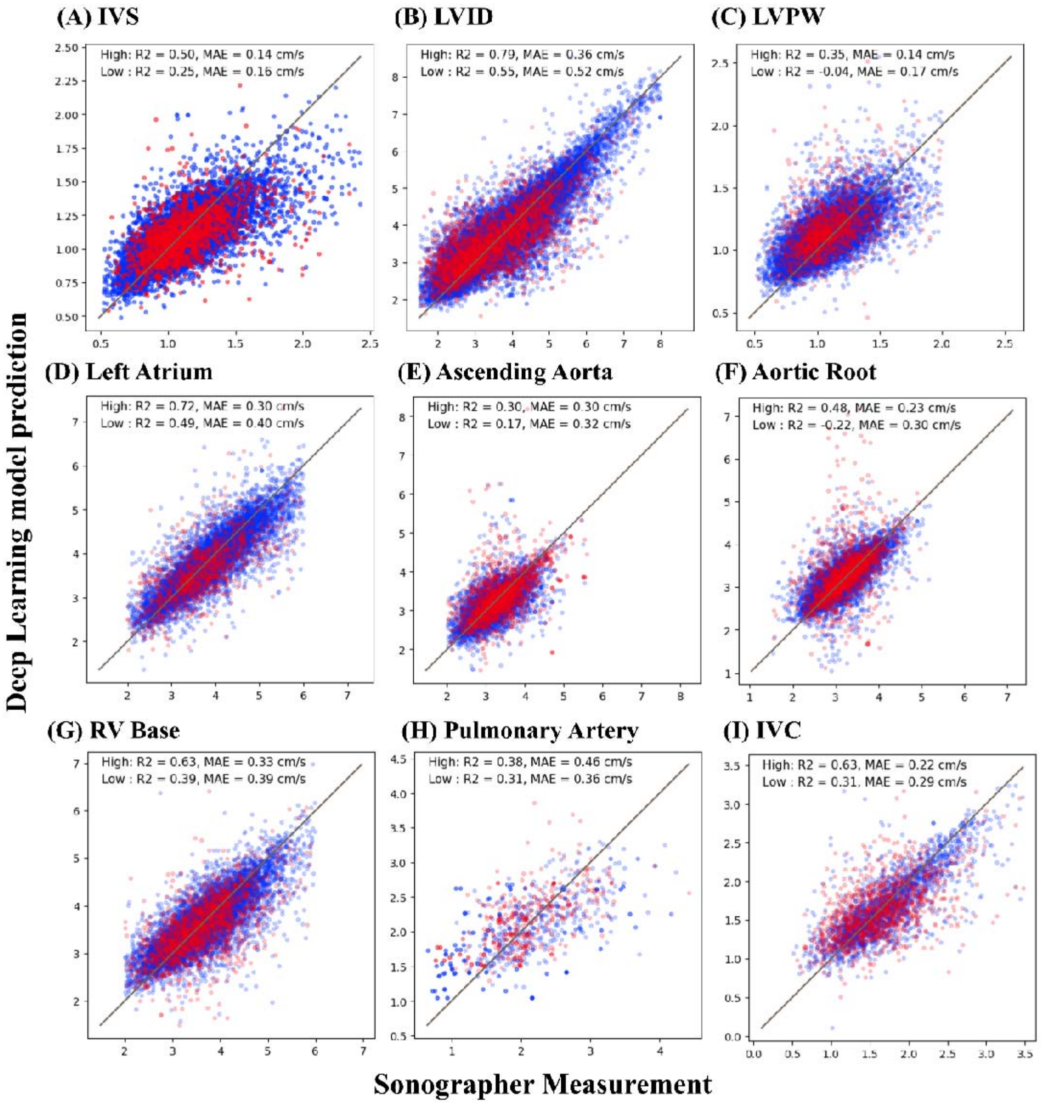

Scatterplot between deep learning model predictions and sonographer annotations for various echocardiographic linear measurements, classified by predicted image quality. Each plot represents a specific measurement, with the x-axis showing the values annotated by the sonographer and the y-axis showing the values predicted by the Deep Learning model. Blue dots indicate higher quality images predicted by image-quality model, and red dots indicate lower quality images predicted by the same model. The coefficient of determination (R2) and mean absolute error (MAE). For a detailed

medRxiv preprint doi: https://doi.org/10.1101/2025.03.18.25324215; this version posted March 19, 2025. The copyright holder for this preprint (which was not certified by peer review) is the author/funder, who has granted medRxiv a license to display the preprint in perpetuity.
It is made available under a CC-BY 4.0 International license .

explanation of echocardiography parameter abbreviations, refer to Supplemental Table 1.

Supplemental Figure 3: Correlation between Deep Learning Model and Sonographer Annotations for Echocardiographic Doppler Measurements Classified by Predicted Image-Quality

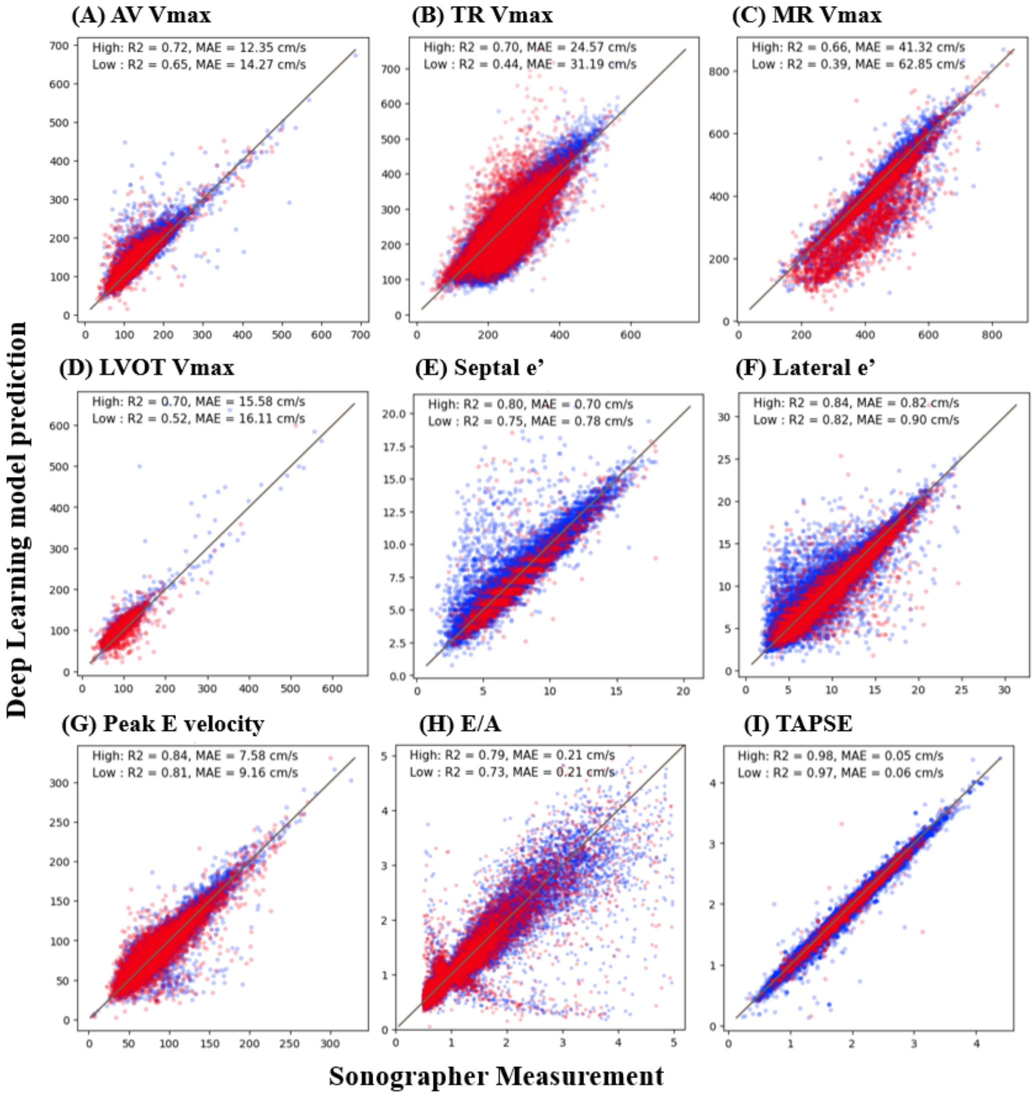

Scatterplot between deep learning model predictions and sonographer annotations for various echocardiographic Doppler measurements, classified by predicted image quality. Each plot represents a specific measurement, with the x-axis showing the values annotated by the sonographer and the y-axis showing the values predicted by the Deep Learning model. Blue indicates higher quality images predicted by image-quality model, and red indicates lower quality images predicted by the same model. The coefficient of

medRxiv preprint doi: https://doi.org/10.1101/2025.03.18.25324215; this version posted March 19, 2025. The copyright holder for this preprint (which was not certified by peer review) is the author/funder, who has granted medRxiv a license to display the preprint in perpetuity.
It is made available under a CC-BY 4.0 International license .

determination (R2) and mean absolute error (MAE). For a detailed explanation of echocardiography parameter abbreviations, refer to Supplemental Table 1.

medRxiv preprint doi: https://doi.org/10.1101/2025.03.18.25324215; this version posted March 19, 2025. The copyright holder for this preprint (which was not certified by peer review) is the author/funder, who has granted medRxiv a license to display the preprint in perpetuity.
It is made available under a CC-BY 4.0 International license .

Supplemental Figure 4: Model Performance and Agreement of EchoNet-Measurement in the external test dataset with Study-level ground truth value

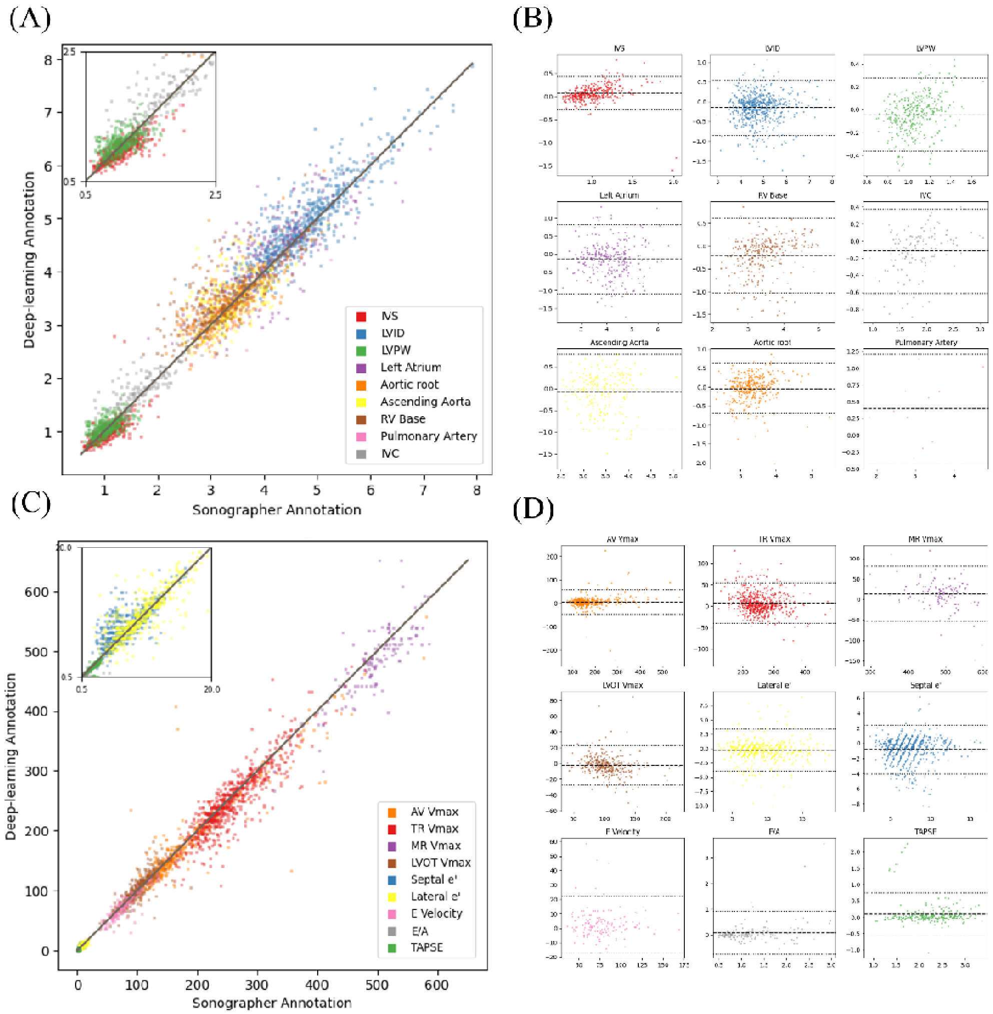

(A and C) Scatter plots comparing deep learning model predictions with sonographer annotations for linear measurements (A) and Doppler echocardiography parameters (C). (B and D) Bland-Altman plots for each parameter in the linear measurement group (B) and Doppler echocardiography parameters (D). All metrics including coefficient of determination (R2), intraclass correlation coefficients (ICC), mean absolute error (MAE), bias and limits of agreement are described in **Supplemental Table 3** (Stanford Health Care dataset with study-level ground truth measurement value), respectively. Refer to Table 1 for a detailed explanation of echocardiography parameter abbreviations, Figure 2 for the explanation of figure legend and Bland-Altman plot. In Bland-Altman Plot (B and D), gray and black lines indicate bias and limits of agreement.

medRxiv preprint doi: https://doi.org/10.1101/2025.03.18.25324215; this version posted March 19, 2025. The copyright holder for this preprint (which was not certified by peer review) is the author/funder, who has granted medRxiv a license to display the preprint in perpetuity.
It is made available under a CC-BY 4.0 International license .

Supplemental Figure 5: Representative Cases of High Measurement Discrepancies Analysis in Echocardiographic Measurements

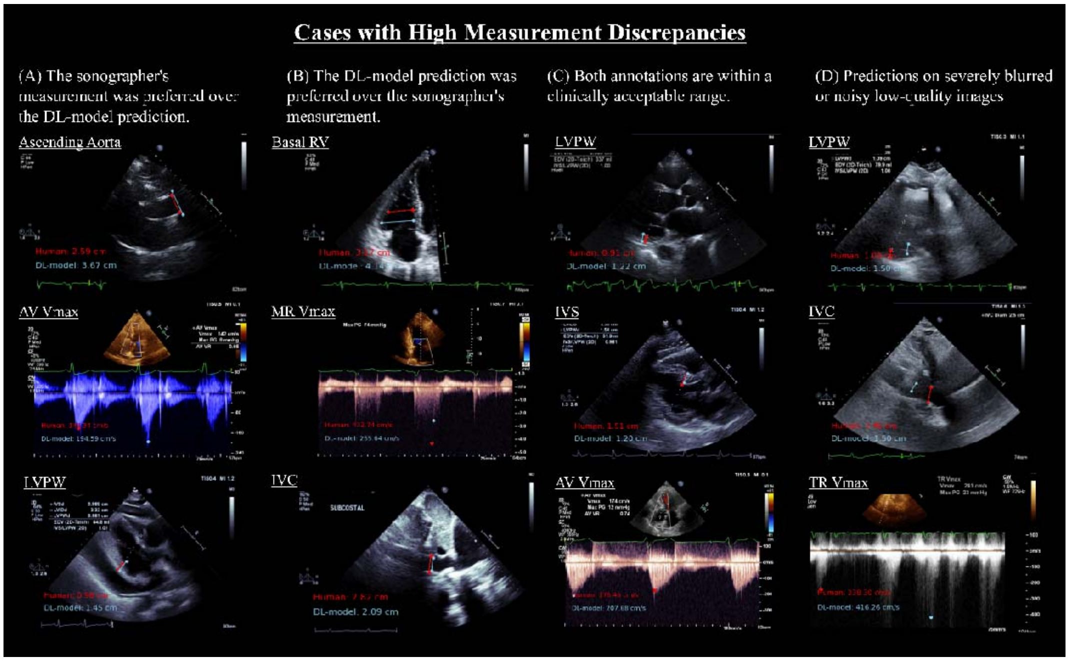

Representative echocardiography images of high measurement discrepancies analysis. Reviewed images were categorized into four categories; (A) cases where the reviewer considered that the sonographer's measurement was preferred over the deep-learning (DL) model prediction, (B) cases where the DL model's prediction was preferred over the sonographer's measurement, (C) cases where both annotations (sonographer and DL model) were within a clinically acceptable range despite measurement variability, (D) cases where the echocardiographic images were severely noisy or blurred, considered inappropriate images for the DL-model input. Red texts and dots indicate sonographer measurements, and blue texts and dot show the DL model's measurements. For a detailed explanation of echocardiography parameter abbreviations, refer to Supplemental Table 1.

medRxiv preprint doi: https://doi.org/10.1101/2025.03.18.25324215; this version posted March 19, 2025. The copyright holder for this preprint (which was not certified by peer review) is the author/funder, who has granted medRxiv a license to display the preprint in perpetuity.
It is made available under a CC-BY 4.0 International license .

Supplemental Video 1: Beat-to-beat Linear Measurement Parameters Prediction

Legend: This video demonstrates (A through I) the deep learning model's beat-to-beat predictions for multiple linear measurement parameters across echocardiographic images. For a detailed explanation of echocardiography parameter abbreviations, refer to Supplemental Table 1.

Supplemental Video 2: Demo for High-Throughput TR Vmax Measurement and Annotation Pipeline for CW Doppler Waveform Prediction

Legend: This interface developed using Gradio demonstrates a process for uploading multiple DICOM images containing TR Vmax measurements and performing high-throughput annotations using an automated pipeline. Users can upload multiple DICOM files and these are then processed to annotate the predicted points and calculate the TR Vmax values. The outputs including annotated Doppler images and extracted TR Vmax values are displayed in a results table and can be exported as a CSV file.

Supplemental Methods

For the development of the quality-control model for the linear measurement group, a video-based convolutional neural network (R2+1D) was used with standardized input videos of 112 × 112 pixel for linear measurement group. For the Doppler image quality-control model, an image-based model (DenseNet) was employed with 426 × 1024 still image inputs. A dataset of 31,506 manually curated videos (9.8% were classified as poor quality videos) from 21,035 patients and 29,232 still images (21.5% were classified as poor images or noisy images) from 20,951 patients in CSMC was split 8:1:1 ratio by patient medical record number and all videos and images were classified as low-quality or high-quality images (representative ground truth images in **Supplemental Figure 2**). Image quality was evaluated by two cardiologists (Y.S and C.B.R). The model was trained to minimize binary cross-entropy loss using the AdamW optimizer. The initial learning rate was set to 1x10-5, with a batch size of 10 for the video model and 24 for the image model, over the course of 50 epochs with an early stopping setting. Model performance was evaluated in the held-out dataset, achieving an AUROC of 0.984 in the linear measurement group and an AUROC of 0.912 in the Doppler measurement group.

medRxiv preprint doi: https://doi.org/10.1101/2025.03.18.25324215; this version posted March 19, 2025. The copyright holder for this preprint (which was not certified by peer review) is the author/funder, who has granted medRxiv a license to display the preprint in perpetuity.
It is made available under a CC-BY 4.0 International license .

Further, a classification model was developed using a similar procedure to exclude cases where annotated point was plotted on a’ during septal or lateral e' prediction. Datasets were manually reviewed and finally datasets annotated with only e' (n=7962) or only a' (n=2128) were prepared. A classification model was then built using DenseNet and abovementioned hyperparameters and image sizes. This model achieved an AUROC of 0.965 on a test dataset split by patient in an 8:1:1 ratio, and the optimal cutoff value was determined using the Youden Index. Following the inference of septal e' and lateral e' prediction, data plotted as a' were removed by passing them through this model.
